# To use stroke 911 to improve stroke awareness for countries where 911 is used as an emergency phone number

**DOI:** 10.1111/cns.13931

**Published:** 2022-08-04

**Authors:** Renyu Liu, Jing Zhao, Xiaobin Li, Steven Messe, Marc Fisher, Anthony Rudd

**Affiliations:** ^1^ Department of Anesthesiology and Critical Care Perelman School of Medicine at the University of Pennsylvania Philadelphia Pennsylvania USA; ^2^ Department of Neurology Perelman School of Medicine at the University of Pennsylvania Philadelphia Pennsylvania USA; ^3^ Department of Neurology, Minhang Hosptial Fudan University Shanghai China; ^4^ Internal Medicine and President Everest Medical Group Philadelphi Pennsylvania USA; ^5^ Department of Neurology, Beth Israel Deaconess Medical Center Harvard Medical School Boston Massachusetts USA; ^6^ Stroke Medicine Kings College London London UK

**Keywords:** emergency, FAST, ischemic, prehospital delay, stroke, stroke 911

## 1 INTRODUCTION

**FIGURE 1:**
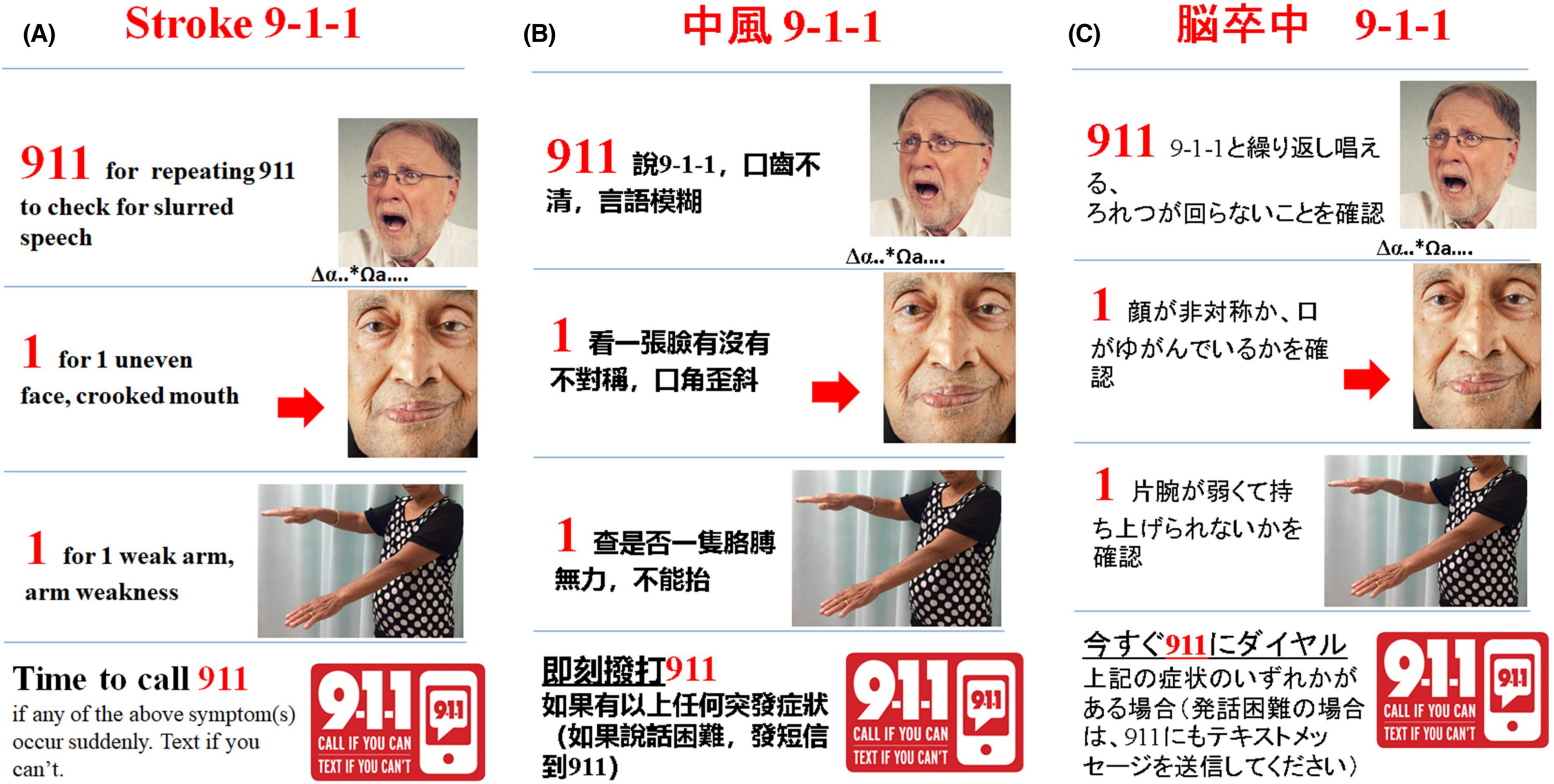
Examples of educational material developed for Chinese people living in the US. (A) The English version, (B) the traditional Chinese version, (C) The Japanese version translated by Dr. Akira Nishisaki at the Children's Hospital of Philadelphia.

In 2020, stroke ranked as the third cause of death from non‐communicable diseases (except injury) in the United States.^1^ World Health Organization data from 2019 indicated that stroke is the number two cause of death worldwide. Stroke is a preventable and now eminently treatable disease. However, partly due to poor awareness and significant prehospital delay, the majority of people with ischemic stroke arrive too late to receive available treatments like thrombolysis and thrombectomy, as these treatments are available only within certain time frame after a stroke. The acronym FAST (*Face, Arm, Speech*, and *Time*) is the most popular and effective tool for stroke awareness and recognition, predominantly for the English‐speaking populations. However, as with any acronym, FAST does not effectively translate in a way for easy remembering for non‐English speakers and the meaning of “fast action” may be lost. While English is the most spoken language followed by Chinese worldwide, only 400 million are native English speakers among approximately 7.8 billion people. To overcome the language barrier, we have proposed novel strategies that use emergency phone numbers as mnemonic tools for stroke signs and symptoms, including Stroke 120, Stroke 112, and Stroke 911.^2^, ^3^, ^4^ These strategies are based on the core concept of “FAST”. The acceptance and effectiveness of Stroke 120 and Stroke 112 have been demonstrated.^3^, ^5^ Here, we present a modified stroke 911 strategy to improve stroke awareness for countries and regions where 911 is used as an emergency phone number, especially for those whose first native language is not English as indicated in Figure 1.^2^ The major modification is to ask the potential stroke victim to repeat 911 to check speech disturbance in any language instead of asking spelling N‐I‐N‐E to check speech disturbance. The advantages of the system include (1) it is based on the well‐accepted stroke recognition FAST strategy; (2) it overcomes the language barrier since people do not need to remember all of the English words needed for the acronym of FAST; (3) It links the emergency phone number 911 to the common stroke signs and symptoms; and, (4) It can be translated into any language for educational purposes without losing its core meaning for stroke recognition and immediate action.

## 2 TO TEST THE STRATEGY IN THE UNITED STATES TO IMPROVE STROKE AWARENESS IN MINORITIES IN THE UNITED STATES

A recent study indicated that Asian American patients manifested more severe ischemic strokes, were less likely to receive “clot buster” therapies such as IV tPA and had worse functional outcomes than white patients.^6^ For those with a verified onset to arrival time, Asian American patients took longer on average to arrive at the hospital after ischemic stroke onset (mean, 554.3 min) than white patients (mean, 471.5 min). Also, a lower percentage of Asian American patients than white patients arrived within 4.5 h from stroke onset (51.5% vs. 57.5%), which is the time window most patients could be treated with a thrombolytic. Stroke treatment is exquisitely time‐sensitive. Delays to arrival and assessment are associated with a lower likelihood of being treated, and a lower likelihood of a good outcome among those who do receive treatment.^7^, ^8^, ^9^ Thus, delay in hospital arrival is one of the main reasons that Asians have worse outcomes from stroke than white patients. Racial and ethnic minority groups have been shown to have less knowledge about stroke, which could lead to disparities in timely stroke hospital presentation. Cultural tailoring of stroke education may, therefore, be an effective approach to improve stroke outcomes. The estimated number of Asian Americans is about 24 million. Chinese, Indian, and Filipino Americans make up the largest share of the Asian American population with 5, 4.3, and 4 million people, respectively, representing a diversity of languages. Among these different languages, there were 2.8 million people (age five and older) who spoke one of the Chinese dialects at home. Chinese is the third most common language in the United States. Based on the US Department of Health and Human Services Office of Minority Health, 42.0% percent of Chinese over the age of five who live in the United States do not speak English very well. Therefore, it is reasonable to first target the Chinese community using the Chinese language and a culture‐adapted approach to improve stroke awareness and promote the immediate action of calling 911 to reduce stroke‐related mortality and morbidity. As indicated in Figure 1B,C, we have translated the tool into both simplified Chinese and traditional Chinese. The tool has received enthusiastic support from our colleagues with native language capability to translate this tool to other languages. These include Japanese by Akira Nishisaki, MD; Korean by Si Ju Kim, CRNA, Vietnamese by Bao Ha, MD; Hmong by Kia Lor, MD; Hindi by Deepa Chen. You may contact Dr. Renyu Liu to obtain these materials or help us to translate these materials for other languages.

**FIGURE 2:**
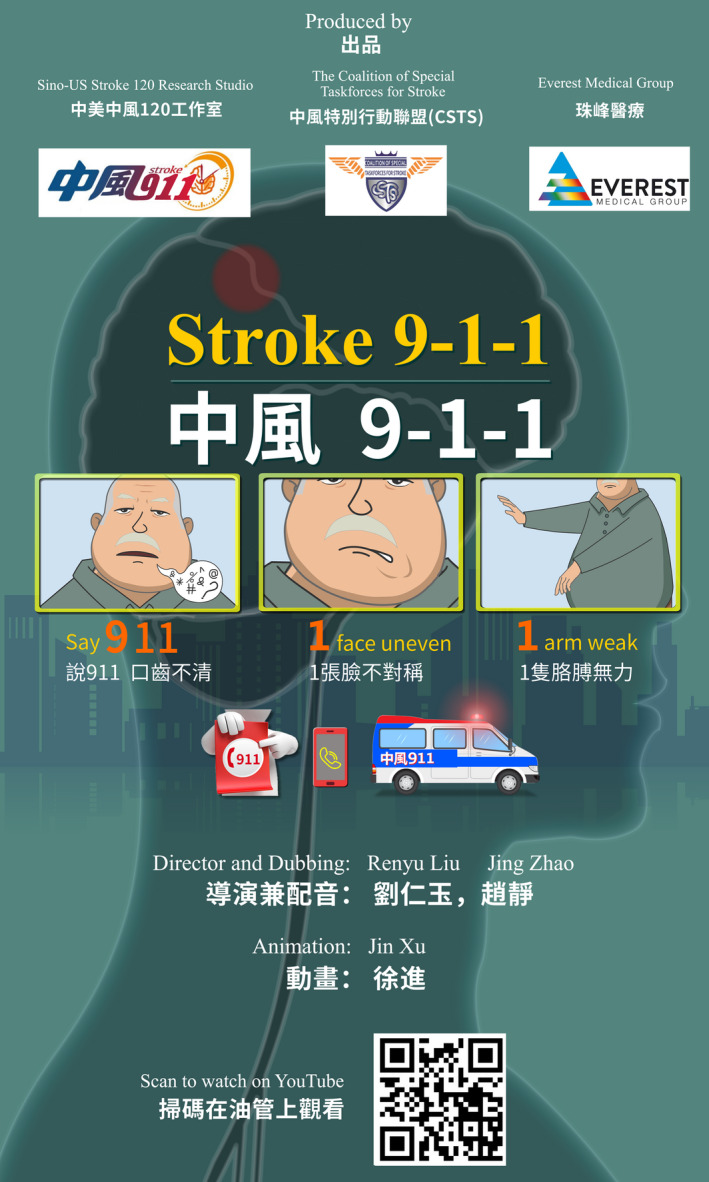
The video poster introduction with QR code to access the YouTube video. The video is produced in Chinese with Chinese, English, and Korean captions at the moment. The Korean caption for Koreans living in the United States was added by Esther Kim, MD, who is currently an anesthesia resident at the Hospital of University of Pennsylvania. YouTube link is as follows: https://youtu.be/c_e0xfuxJe0

To help implement the educational program we produced a short video for Stroke 911, which is available to the public via YouTube.^10^ The poster introduction is presented in Figure 2. After demonstrating the effectiveness in the US Chinese community we plan to expand to other minority communities by using a similar approach, adapting it to their unique languages and cultures, focusing on those communities with the lowest English proficiency. The proposed approach could potentially be implemented as professional guidelines for policy‐making purposes to improve the health of minority populations and reduce disparity.

## 3 EXPANSION TO OTHER COUNTRIES AND REGIONS WHERE 911 IS USED AS AN EMERGENCY PHONE NUMBER

Based on the World Population Review, 48 countries and regions including USA use 911 to call an emergency ambulance. Similar to the USA, Canada has a diversity of native languages. More than 40% of Canadian's native language is not English. Therefore, it is highly possible that this strategy could be used in other countries and regions with a simple translation. The Coalition of the Special Taskforces for Stroke (CSTS) will make the effort to achieve this. We will make all the educational materials available online through CSTS official website.

## FUNDING INFORMATION

National Natural Science Foundation of China; CIHR, Grant/Award Number: 81973157, PI: JZ. Funding from the University of Pennsylvania; Grant/Award Number: CREF‐030, PI: RL.

## CONFLICT OF INTEREST

All authors have no conflict of interest to declare.

## Data Availability

The data that support the findings of this study are available from the corresponding author upon reasonable request.

## References

[1] Murphy S , Kochanek K , Xu J , Arias E . Mortality in the united states, 2020 NCHS Data Brief No 427 2021 https://www.cdc.gov/nchs/data/databriefs/db427.pdf 34978528

[2] Liu R , Fisher M , Rudd A , Zhao J . Speech disturbance plays critical role in stroke recognition during covid‐19 pandemic. CNS Neurosci Ther. 2021;27:267‐269. 33452869 10.1111/cns.13608 PMC7871789

[3] Zhao J , Eckenhoff MF , Sun WZ , Liu R . Stroke 112: a universal stroke awareness program to reduce language and response barriers. Stroke. 2018;49:1766‐1769. 29925649 10.1161/STROKEAHA.118.021729 PMC6034704

[4] Zhao J , Liu R . Stroke 1‐2‐0: a rapid response programme for stroke in China. Lancet Neuro. 2017;16:27‐28. 10.1016/S1474-4422(16)30283-6 PMC5578829 28029517

[5] Zhao J , Li X , Liu X , et al. Changing the strategy and culture of stroke awareness education in China: implementing stroke 1‐2‐0. Stroke Vasc Neurol. 2020;5:374‐380. 32350059 10.1136/svn-2019-000324 PMC7804060

[6] Song S , Liang L , Fonarow GC , et al. Comparison of clinical care and in‐hospital outcomes of asian american and white patients with acute ischemic stroke. JAMA Neuro. 2019;76:430‐439. 10.1001/jamaneurol.2018.4410 PMC6459126 30667466

[7] Saver JL , Goyal M , van der Lugt A , et al. Time to treatment with endovascular thrombectomy and outcomes from ischemic stroke: a meta‐analysis. JAMA. 2016;316:1279‐1288. 27673305 10.1001/jama.2016.13647

[8] Saver JL , Fonarow GC , Smith EE , et al. Time to treatment with intravenous tissue plasminogen activator and outcome from acute ischemic stroke. JAMA. 2013;309:2480‐2488. 23780461 10.1001/jama.2013.6959

[9] Powers WJ , Rabinstein AA , Ackerson T , et al. Guidelines for the early management of patients with acute ischemic stroke: 2019 update to the 2018 guidelines for the early management of acute ischemic stroke: a guideline for healthcare professionals from the american heart association/american stroke association. Stroke. 2019;50:e344‐e418. 31662037 10.1161/STR.0000000000000211

[10] Liu R , Zhao J , Li X . Stroke 911 video (animation). 2022 https://youtu.Be/c_e0xfuxje0

